# Shedding light on eating disorders in adolescents with type 1 diabetes: insights and implications

**DOI:** 10.1007/s00431-025-06081-0

**Published:** 2025-03-31

**Authors:** Sohier Yahia, Nanees A. Salem, Salwa Tobar, Zahraa Abdelmoneim, Ahmed Magdy Mahmoud, Wafaa Laimon

**Affiliations:** 1 Department of Pediatrics, Genetics Unit, Faculty of Medicine, Mansoura University, Mansoura, Egypt; 2 Department of Pediatrics, Endocrinology Unit, Faculty of Medicine, Mansoura University, Mansoura, Egypt; 3 Department of Psychiatry, Faculty of Medicine, Mansoura University, Mansoura, Egypt; 4 Mansoura University Children Hospital, El-Gomhoria St, 35516, Box 50, Mansoura, 53355 Egypt

**Keywords:** Eating disorders, Eating behaviors, Type 1 diabetes

## Abstract

Eating disorders (EDs) are complex medical conditions that pose a considerable health burden for individuals with type 1 diabetes mellitus (T1DM). EDs in individuals with T1DM are linked to poor metabolic control, which heightens the risk of diabetes complications. Consequently, regular screening for EDs is essential. This study investigates the prevalence of EDs in adolescents with T1DM, investigating the associations with diabetes duration, pubertal stage, glycemic control, and diabetes-related complications. In this cross-sectional study, 350 adolescents (155 males, 195 females) with T1DM, aged 12–18, were recruited from Mansoura University Children’s Hospital. Participants completed the Diabetes Eating Problem Survey-Revised (DEPS-R) questionnaire, with scores ≥ 20 prompting clinical interviews to confirm EDs. Clinical data, including HbA1c, BMI, and body composition, were analyzed. Socioeconomic status (SES) and family factors were assessed. The prevalence of EDs was 22.6%, including other specified feeding or eating disorders (OSFED) (68.4%), binge eating (11.4%), bulimia nervosa (7.6%), avoidant restrictive (7.6%), and anorexia nervosa (5.1%). Binary logistic regression analysis showed that the significant predictors of ED in the study cohort were diabetes duration (OR = 1.75 (1.66–1.84), *p* < 0.001), and HbA1c (OR = 5.94 (2.4–14.6), *p* < 0.001). *Conclusions*: Adolescents with EDs had higher (SES), more family conflicts, longer diabetes duration, and were more prone to diabetic nephropathy and poor glycemic control. Screening for EDs is recommended from pre-adolescence through early adulthood.

**Table:** 

**What Is Known:** *• Adolescents with T1DM are more vulnerable to develop EDs compared to their peers without T1DM* **What Is New:** *• The DEPS-R and DSM-V were useful clinical tools for screening and for diagnosis of EDs respectively among adolescents with T1DM* *• We advise to screen for EDs in adolescents with T1DM who aged around 13.6 years, at Tanner stage 3, with duration of T1DM >5 years, and/or with HbA1c >7.5%*

**What Is Known:**

*• Adolescents with T1DM are more vulnerable to develop EDs compared to their peers without T1DM*

**What Is New:**

*• The DEPS-R and DSM-V were useful clinical tools for screening and for diagnosis of EDs respectively among adolescents with T1DM*

*• We advise to screen for EDs in adolescents with T1DM who aged around 13.6 years, at Tanner stage 3, with duration of T1DM >5 years, and/or with HbA1c >7.5%*

## Introduction

Eating disorders (EDs) are complex medical conditions that represent a significant morbidity among people with type 1 diabetes mellitus (T1DM) [1, 2]. The prevalence of EDs diagnosed according to the Diagnostic and Statistical Manual of Mental Disorders (DSM), and disordered eating behaviors (DEBs), that does not merit a formal EDs diagnosis, has been reported to be significantly higher among people with T1DM [3]. This can be explained by multiple factors related to diabetes management as the emphasis on diet and glycemic targets, the focus on achievement of ideal body mass index (BMI), insulin-related weight gain, and associated body image dissatisfaction [4]. Moreover, intentional insulin omission for weight reduction is a unique diabetes-related DEB [5].

EDs and DEBs typically begin during adolescence and early adulthood [1]. EDs are associated with poor metabolic control and increase the risk for developing short and long-term diabetic complications [3, 4]. Therefore, screening for EDs should begin in pre-adolescence and continue through early adulthood, as many DEBs begin during the transition to adolescence and may persist for years.

The current study aimed to investigate the prevalence of EDs among adolescents with T1DM in relation to onset and duration of diabetes, pubertal stage, level of glycemic control, and diabetes-related complications.

## Subjects and methods

### Study design

This cross-sectional study included three hundred and fifty adolescents with T1D recruited from the Pediatric Diabetes Clinic, Mansoura University Children’s Hospital (MUCH), Egypt, during the period from December 2021 to June 2023.

### Participants

The study included adolescents diagnosed with T1DM according to the International Society of Pediatric and Adolescents Diabetes (ISPAD) 2018 Guidelines [6], aged 12–18 years and maintained on daily insulin therapy with basal-bolus regimen for at least 1 year. Adolescents with coeliac disease, gastrointestinal problems, autoimmune thyroiditis, T2DM, monogenic diabetes, and those with history of major psychiatric illness (including, autism, attention deficit hyperreactivity disorders (ADHD), bipolar disorders, major depression, and schizophrenia) were excluded. Our rationale for this exclusion was to reduce confounding effects and focus on primary ED detection in a T1DM population.

## Methods

### Clinical assessment

All included subjects underwent detailed medical history including diabetes onset, duration, insulin daily dose (U/kg/day), hospital admission, diabetic ketoacidosis (DKA), and hypoglycemia frequency. The socioeconomic status (SES) was evaluated using an Egyptian-validated Arabic SES scale. It is a seven-domain scale with a total score of 84. According to this scale, the SES is either very low, low, middle, or high [7]. Family troubles were assessed through clinical history taking, focusing on factors such as parental relationship status (e.g., divorce or separation) and the presence of domestic violence.

Thorough clinical examination with emphasis on anthropometric measures and body mass index (BMI) measured as kilogram per square meter.

### Psychological assessment

Participants completed the diabetes eating problem survey-revised (DEPS-R) questionnaire in a paper-based model with a diabetes nurse who clarified any item that needed more understanding. It is a 16-item structured questionnaire with a six-point Likert scale where “0” represents “never,” and “5” represents “always.” The overall score ranges from zero to 80, with a higher score indicating more DEBs. DEPS-R score cutoff point ≥ 20 indicates a high risk for DEBs [8, 9]. Participants who scored ≥ 20 were invited to a clinical interview for diagnosis of EDs using DSM, Fifth Edition for feeding and eating disorders [10].

### Laboratory Investigations and body composition evaluation 

The mean glycosylated hemoglobin (HbA1c) in the last year of follow-up was recorded. Measurement of albumin/creatinine ratio in a fresh morning urine sample by enzyme-linked immunosorbent assay (ELISA) was used to screen for diabetic nephropathy. Body composition measurements were obtained by bio-impedance technique using Tanita BC-418MA body composition analyzer (Tanita Corp., Tokyo, Japan) according to the manufacturer’s instructions. It provides measurements for total and segmental body composition.

### Statistical analysis

Data analysis was performed by SPSS software, version 24 (SPSS Inc., PASW statistics for windows version 24. Chicago: SPSS Inc.). Qualitative data were described as frequencies and percentages. Quantitative data were described using median (minimum and maximum) (interquartile range) for non-normally distributed data and mean ± standard deviation for normally distributed data after testing normality using the Kolmogorov–Smirnov test. Significance of the obtained results was judged at the (0.05) level. The chi-square test was used to compare qualitative data between groups as appropriate. Mann–Whitney *U* and Kruskal–Wallis tests were used to compare between 2 studied groups and more than 2 studied groups, respectively for non-normally distributed data. The Student *t* test was used to compare 2 independent groups for normally distributed data. The one-way ANOVA test was used to compare more than 2 independent groups with post hoc Tukey test to detect pair-wise comparison. Binary logistic regression was done to detect predictors of eating disorders among studied cases using Enter technique and with calculation of odds ratio.

## Results

A total of 350 adolescents with T1DM (155 males and 195 females) were enrolled in the study. The mean age of participants was 13.86 ± 1.95 years, with median diabetes duration of 5 (3–14) years. The mean HbA1c was 8.2 ± 1.25%, and the average total daily insulin dose was 1.55 ± 0.45 unit/kg/day.

**Fig. 1:**
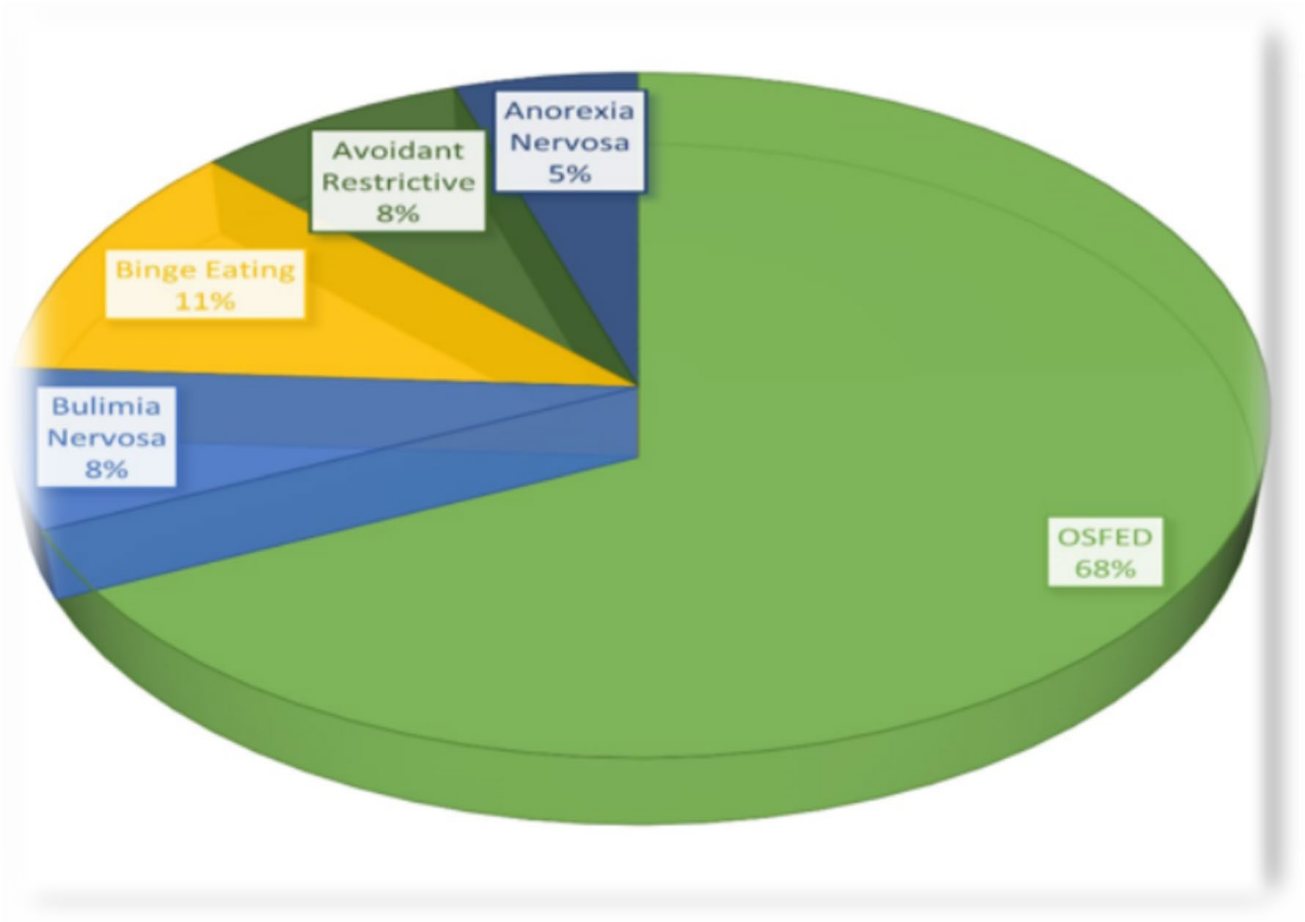
Eating disorders among the studied adolescent with type 1 diabetes. OSFED, specified feeding or eating disorder

The prevalence of EDs among the study cohort was 22.6% (*n* = 79). The distribution of specific EDs was as follows: 68.4% other specified feeding or eating disorder (OSFED), 11.4% binge eating, 7.6% bulimia nervosa (BN), 7.6% avoidant restrictive, and 5.1% anorexia nervosa (AN) (Fig. 1).

### Sociodemographic and clinical characteristics

A significant association was observed between SES and the presence of EDs, with a higher prevalence of EDs among participants from high SES backgrounds compared to those without EDs (48.1% versus 10.7%, *p* = 0.04). Additionally, family conflicts were significantly more frequent in adolescents with EDs compared to those without EDs (89.9% versus 17.7%, *p* < 0.001) (Table 1).

**Table 1:** Clinicodemographic characteristics of the study subjects

	Total study cohort *n* = 350	Non-ED group (DEPS-R < 20) *n* = 271	ED group (DEPS-R ≥ 20) *n* = 79	Test of significance
Age (years)	13.86 ± 1.95	13.92 ± 1.93	13.67 ± 2.01	*t* = 0.982 *p* = 0.327
Sex				
Male	155 (44.3%)	116 (42.8%)	39 (49.4%)	*χ*^2^ = 1.07
Female	195 (55.7%)	155 (57.2%)	40 (50.6%)	*p* = 0.301
Residence				
Urban	83 (23.7%)	58 (21.4%)	25 (31.6%)	*χ*^2^ = 3.55
Rural	267 (76.3%)	213 (78.6%)	54 (68.4%)	*p* = 0.06
Family troubles				
Yes	119 (34%)	48 (17.7%)	71 (89.9%)	*χ*^2^ = 141.94
No	231 (66%)	223 (82.3%)	8 (10.1%)	*p* < 0.001*
SES				
Low	152 (43.4%)	126 (46.5%)	26 (32.9%)	*χ*^2 MC^ = 6.33
Middle	131 (37.4%)	116 (42.8%)	15 (19.0%)	*p* = 0.04*
High	67 (19.1%)	29 (10.7%)	38 (48.1%)	
BMI (kg/m^2^)	22 ± 0.85	22.01 ± 3.91	22.11 ± 5.25	*t* = 0.182 *p* = 0.856
Age at T1D onset (years)	8 (5–14)	8 (6–11)	11 (9–12)	Z = 1.99 *p* = 0.042*
Duration of diabetes	5 (3–14)	5 (2–6)	6 (3–7)	Z = 2.04 *p* = 0.04*
Total insulin daily dose (unit/kg/day)	1.55 ± 0.45	1.35 ± 0.25	1.58 ± 0.40	*t* = 0.188 *p* = 0.851
Frequency of hypoglycemia				
Yes	87 (24.8%)	67 (24.7%)	20 (25.3%)	*χ*^2^ = 0.015
No	263 (75.2%)	204 (75.3%)	59 (74.7%)	*p* = 0.915
Frequency of DKA Yes No	210 (60%) 140 (40%)	163 (60%) 108 (40%)	57 (72.2%) 22 (27.8%)	*χ*^2^ = 3.78 *p* = 0.052
Frequency of hospital admission				
Yes	220 (62.9)	201 (74.2)	67 (84.8)	*χ*^2^ = 3.86
No	130 (37.1)	70 (25.8)	12 (15.2)	*p* = 0.049*
HbA1c (%)	8.2 ± 1.25	7.8 ± 1.64	8.9 ± 1.05	*t* = 2.122 *p* = 0.038*
Diabetic nephropathy				
Present	115 (32.9%)	73 (26.9%)	42 (53.2%)	*χ*^2^ = 19.07
Absent	235 (67.1%)	198 (73.1%)	37 (46.8%)	*p* = 0.001*

Data is presented as mean ± SD or number (frequency %)

*MC*, Monte Carlo test; *t*, Student *t* test; *χ*^2^, chi-square test; Z, Mann–Whitney *U* test

*Statistically significant

*ED*, eating disorders; *DEPS-R*, diabetes eating problem survey-revised; *DKA*, diabetic ketoacidosis; *HbA1c*, glycated hemoglobin

The median age of T1DM diagnosis was 8 years (range: from 5 to 14 years), with a significantly higher median age of onset among participants with EDs compared to those without (11 years versus 8 years, *p* = 0.042). The median diabetes duration was also significantly longer in those with EDs compared to those without (6 versus 5 years, *p* = 0.04).

### Diabetes-related complications and clinical outcomes

Participants with EDs had a significantly higher frequency of DKA than those without EDs (72.2% versus 60.1%, *p* = 0.052). Additionally, hospital admission rates were significantly higher in the ED group (72.2% versus 60%,* p* = 0.049). There is statistically significant higher prevalence of diabetic nephropathy among those with EDs (53.2% versus 26.9%, *p* = 0.001) (Table 1).

Among female participants, those with EDs had a significantly higher waist circumference than those without (84.95 versus 78.70; *p* = 0.001). A higher frequency of EDs was observed in participants at Tanner stage 3, *p* = 0.04 (Table 2).

**Table 2:** Comparison of blood pressure, anthropometric measurements, and pubertal staging between cases with and without eating disorders

	No eating disorders *n* = 271	Eating disorders *n* = 79	Test of significance
DBP (mm/Hg)	63.47 ± 6.87	64.56 ± 6.76	*t* = 1.24 *p* = 0.214
SBP (mm/Hg)	100.96 ± 7.19	100.76 ± 8.88	*t* = 0.206 *p* = 0.837
Height *Z* score	− 0.727 (− 1.5, 0.602)	− 0.560 (− 1.23, 0.246)	*Z* = 1.73 *p* = 0.083
BMI (kg/m^2^)	22.01 ± 3.91	22.11 ± 5.25	*t* = 0.182 *p* = 0.856
BMI *Z* score	0.437 (− 0.338, 1.32)	0.233 (− 0.385, 1.26)	*Z* = 0.130 *p* = 0.896
Waist circumference/cm			
- Male	77.09 ± 11.52	77.64 ± 12.81	*t* = 0.253, *p* = 0.801
- Female	78.70 ± 9.03	84.95 ± 13.31	*t* = 4.82, *p* = 0.001*
MAC			
- Male	26.16 ± 4.51	26.44 ± 4.58	t = 0.335, *p* = 0.738
- Female	25.86 ± 2.76	26.60 ± 4.48	t = 1.32, *p* = 0.190
Tanner staging; *n* (%)			
1	0 (0)	0 (0)	
2	26 (9.6)	2 (2.5)	*χ*^**2**^ = 4.15, *p* = 0.04*
3	97 (35.8)	38 (48.1)	*χ*^**2**^ = 3.91, *p* = 0.04*
4	64 (23.6)	13 (16.5)	*χ*^2^ = 1.83, *p* = 0.176
5	84 (31)	26 (32.9)	*χ*^2^ = 1.04, *p* = 0.75

Data is presented as mean ± SD

*Z*, Mann–Whitney *U* test; *t*, Student *t* test; *χ*^2^, chi-square test

*BMI*, body mass index; *MAC*, mid-arm circumference; *DBP*, diastolic blood pressure; *SBP*, systolic blood pressure

Total body muscle mass was significantly lower in females with EDS compared to those without EDs (34.1 versus 38 kg, *p* = 0.013) (Table 3).

**Table 3:** Body composition parameters between female cases (*n* = 195) with and without eating disorders

	No eating disorders (DEPS-R < 20) *n* = 155	Eating disorders (DEPS-R ≥ 20) *n* = 40	Test of significance
Total Body fat percent (%)	29 (19.9–34.3)	29.1 (20.33–37.6)	Z = 0.528 *p* = 0.597
Total Body fat mass (kg)	20.25 (17.53–24)	22.2 (19–24.5)	Z = 1.85 *p* = 0.07
Total Body muscle mass (kg)	38 (31.7–43.5)	34.1 (28.28–41.88)	*Z* = 2.49 *p* = 0.013*
Trunk -fat mass (kg)	6.6 (3.7–9.5)	7.15 (2.85–12.2)	*Z* = 0.481 *p* = 0.631

Data is presented as median (IQR)

*Z*, Mann–Whitney *U* test, *statistically significant; *IQR*, interquartile range

*DEPS-R*, diabetes eating problem survey-revised

### Predictors of eating disorders in adolescents with T1DM

DEPS-R score was significantly correlated to diabetes duration (*r* = 0.75, *p* = 0.039) and HbA1c levels (*r* = 0.925, *p* = 0.001) (Table 4).

**Table 4:** Correlation of DEPS-R total score and other variables

	*r*	*p*
Age at time of diagnosis	0.171	0.208
BMI	0.120	0.380
Duration of T1DM in years	0.75	0.039*
HbA1c	0.925	0.001*

*r*, Pearson coefficient

*Statistically significant at *p* ≤ *0.05*

*BMI*, body mass index; *T1DM*, type 1 diabetes mellitus; *HbA1c*, glycosylated hemoglobin

Binary logistic regression analysis identified that longer diabetes duration (OR = 1.75 (1.66–1.84), *p* < 0.001), and higher HbA1c levels (OR = 5.94 (2.4–14.6), *p* < 0.001) as significant predictors of EDs in the study cohort (Table 5).

**Table 5:** Binary logistic regression for prediction of eating disorders among studied cases

	Β	*p* value	Adjusted odds ratio (95% CI)
SES			
- Low			
- Middle	0.603	0.059	1.83 (0.977–3.42)
- High	0.329	0.503	1.39 (0.531–3.64)
Duration of T1DM	0.291	< 0.001*	1.748 (1.664–1.842)
HbA1c	1.78	< 0.001*	5.94 (2.40–14.68)
Tanner staging	0.182	0.353	1.2 (0.817–1.76)
Total body muscle mass (kg)	− 0.082	0.199	0.921 (0.812–1.04)
Overall % predicted = 82.3%			

*CI*, confidence interval; *statistically significant

*SES*, socioeconomic status; *T1DM*, typ1 diabetes mellitus; *HbA1c*, glycosylated hemoglobin

Further logistic regression analysis revealed that adolescents with T1DM duration > 5 years, and those with HbA1c > 7.5% are more likely to develop EDs (OR = 1.2 (0.75 to 3.45), *p* = 0.001) (OR = 2.3 (0.85 to 8.3), *p* = 0.040, respectively) (Table 6).

**Table 6:** Logistic regression analysis of independent predictors for eating disorders in the studied cohort

	***β***	**SE**	***P***	**OR (95%CI)**
**Duration of T1DM (years)**				
**< 5 (r)**				
**> 5**	2.34	0.83	0.001*	1.2 (0.75-3.45)
**HbA1c level (%) **				
**< 7.5% (r)**				
**> 7.5%**	1.29	0.72	0.040*	2.3 (0.85-8.3)
**Tanner Staging**				
**< 2 (r)**				
**>2**	0.86	0.65	0.25	2.42 (1-6.5)
**SES**				
**Low-middle (r)**				
**High**	1.32	0.68	0.31	3.2 (0.90-5.7)

CI, confidence interval; *statistically significant

*T1DM*, typ1 diabetes mellitus; *HbA1c*, glycosylated hemoglobin; *SES*, socioeconomic status

## Discussion

Adolescents with T1DM are more vulnerable to develop EDs compared to their peers without T1DM. EDs typically begin during adolescence and early adulthood. This combination carries higher risk for poor metabolic control and diabetes-related complications [11].

This study included 350 adolescents with T1DM (56.7% were females), with mean age of 13.86 years. Among the study cohort, 22.6% were identified to have EDs (50.6% of them were female).

Prevalence estimates for DEBs are variable between different studies with a wide range from 14.6 to 39% [12–18]. This discrepancy could be attributed to cultural differences, different study designs, variable assessment tools, timing, and sample characteristics among the studied populations.

In the current study, 68.4% of the ED group were identified to have (OSFED). This may be explained by the coexistence of multiple risk factors that contribute to irregular eating pattern in T1DM adolescents leading to DEBs which do not meet the full diagnostic criteria of specific eating disorder group like AN or BN. These risk factors include lifelong insulin therapy, hypoglycemia fear, and social discomfort to have insulin injections in the presence of the others, food preoccupation (e.g., carbohydrate counting), low self-esteem, and depression [18]. Furthermore, Peterson et al. [19] hypothesized that exogenous insulin administration and fluctuations in blood glucose may disrupt natural hunger and satiety in individuals with T1DM. Notably, the same patient may exhibit contradictory eating behaviors, at times consuming excess carbohydrates due to fear of hypoglycemia, while at other times restricting food intake to maintain normal blood glucose levels and normal body weight. This dynamic interplay of behaviors highlights the complexity of EDs in adolescents with T1DM and underscores the need for comprehensive screening and intervention strategies.

The present study revealed that high SES is associated with EDs than T1DM individuals without EDs (48.1% versus 10.7%, respectively). This may be explained by the fact that those with lower SES are less likely to receive proper screening and treatment for EDs, as specialized mental health services are often less accessible due to the high direct and indirect costs of participating in such care [20]. When people with lower SES seek these services, they may face longer wait-times and receive lower levels of care and attention from providers [21]. Similarly, previous researchers showed that ED are so-called diseases of affluence a term used to describe illnesses with elevated prevalence among populations with the highest incomes [22, 23]. Moreover, we found that no significant difference between subtypes of eating disorders regarding SES. In line with our finding, Jones et al. showed the same findings [24].

In our study, a later onset of T1DM and a longer duration of the disease were significantly more common in the ED group.

Takii and his colleagues found out that older age at diabetes onset, in particular during pubertal age with hormonal changes and gain in weight and fat mass may be associated with a greater risk for ED especially in female patients [25].

The present study showed that there is a statistically significant higher frequency of eating disorders among adolescents with T1DM in Tanner staging 3 versus statically significant lower frequency of eating disorders in Tanner staging 2. In line with our findings, prospective and retrospective reports suggest that earlier pubertal timing is associated with elevated rates of eating disorders and bulimic-related symptomatology [26–28].

In our study, DKA frequency was found to be significantly higher in EDs cohort. This copes with what was reported that adolescents and young adults with combined T1DM and EDs have more than triple the risk of DKA and nearly sixfold increased risk of death compared with their peers without EDs [29].

In the present study, females with EDs were found to have higher waist circumference and lower muscle mass. This can be explained by insulin omission as a weight controlling means (Diabulimia) which leads to fat redistribution with central adiposity. Chronic hyperglycemia due to suboptimal insulin use can lead to muscle breakdown. Additionally, T1DM adolescents with DEBs may experience low self-esteem with less interest in physical activity which further contribute to fat accumulation and muscle loss.

### Clinical implication

Adolescents with T1DM are at higher risk of EDs and consequently for higher rates of diabetes-related morbidity and mortality. In the light of our study, it is advised to screen for EDs in individuals with T1DM around the age of 13.6 years and/or Tanner stage 3, particularly in those with diabetes duration > 5 years and/or HbA1c > 7.5%.

### Strength and limitations

In the current study, the dual use of DEPS-R for screening and DSM-V for diagnosis ensures that both clinical and subclinical EDs have been identified. Moreover, this study included a large sample size and captures a range of variables, such as SES, pubertal stage, and glycemic control, enabling a multidimensional analysis of risk factors.

The limitations of this study include being a single-center study with a cross-sectional design which lacks the follow-up or longitudinal dimension and limits causal interferences. Future longitudinal multicenter studies are needed to clarify the directionality of this association and ensure the consistency of results. Body image concerns and family conflicts were not assessed using standardized tools. This presents an important area for future research.

## Conclusion

Eating disorders are prevalent among adolescents with type 1 diabetes, and those with eating problems showed higher frequency of DKA. Diabetes duration more than 5 years and HbA1c > 7.5% have been identified as risk factors for EDs in adolescents with T1DM.

## Data Availability

No datasets were generated or analysed during the current study.

## Abbreviations

BMI: Body mass index
DEBs: Disordered eating behaviors
DEPS-R: Diabetes eating problem survey-revised
DKA: Diabetic ketoacidosis
DSM: Diagnostic and Statistical Manual
EDs: Eating disorders
ELISA: Enzyme-linked immunosorbent assay
HbA1c: Glycosylated hemoglobin
ISPAD: International Society of Pediatric and Adolescents Diabetes
MUCH: Mansoura University Children’s Hospital
OSFED: Other specified feeding or eating disorder
SES: Socioeconomic status
T1DM: Type 1 diabetes mellitus
